# Supplementary material for: An ATR-PrimPol pathway confers tolerance to oncogenic KRAS-induced and heterochromatin-associated replication stress
Source: Nat Commun. 2023 Aug 17;14:4991. doi: 10.1038/s41467-023-40578-2 (PMC10435487; doi:10.1038/s41467-023-40578-2)
Supplement: Supplementary file 1 — Supplementary Information [file 41467_2023_40578_MOESM1_ESM.pdf]

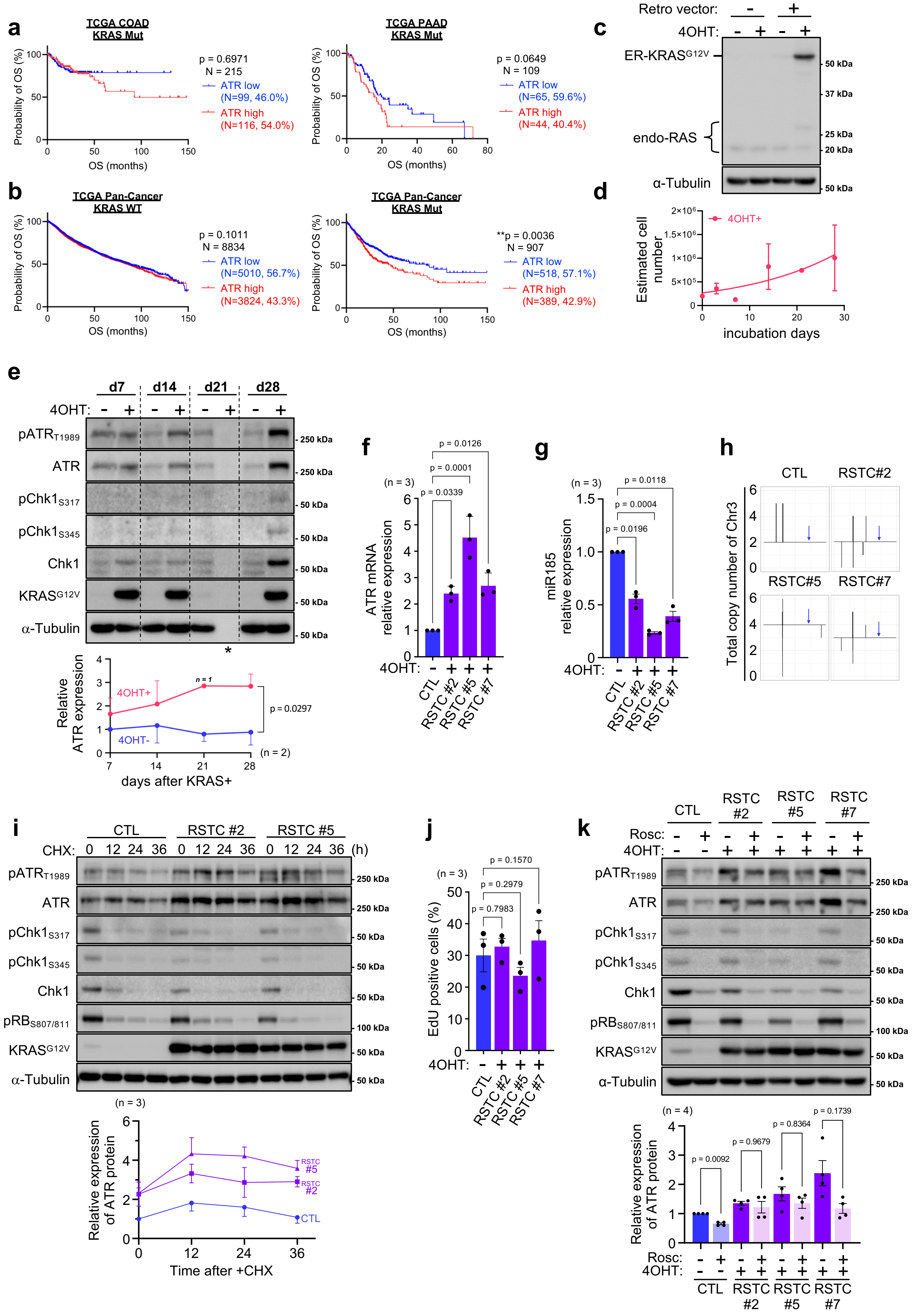

Igarashi et al. Supplementary Figure 1

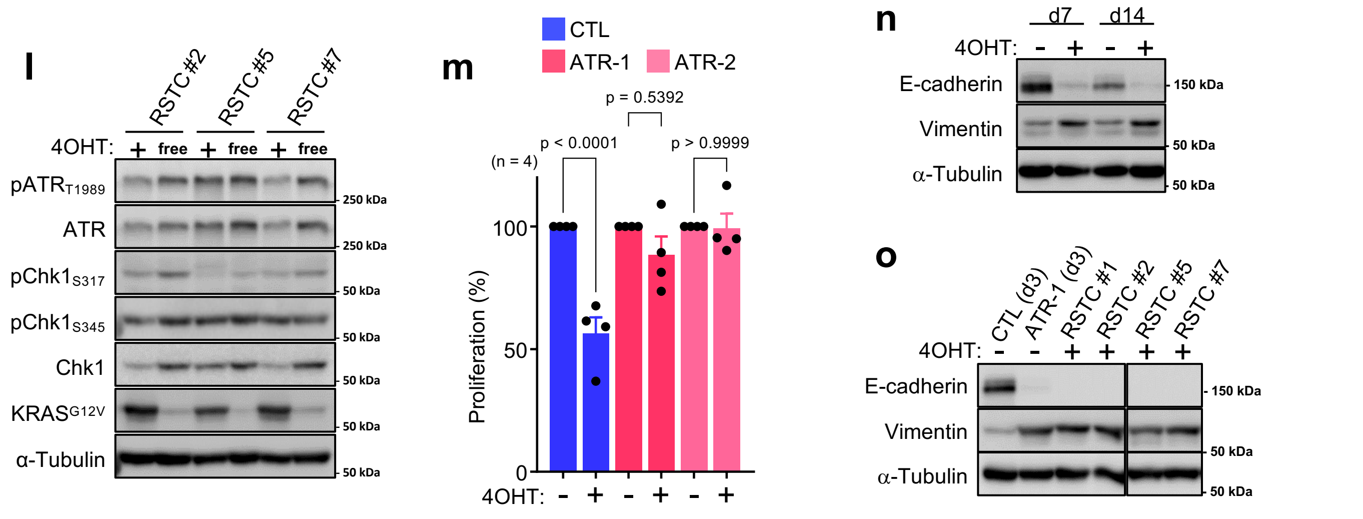

Supplementary Figure 1.

a Overall survival (OS) according to ATR mRNA expression from 215 of colon adenocarcinoma (COAD) and 109 of pancreatic adenocarcinoma (PAAD) patients harboring $KRAS^{Mut}$ were analyzed. Log-rank p values are shown. **b** OS according to ATR mRNA expression from 9741 of pan-cancer patients harboring $KRAS^{WT}$ and $KRAS^{Mut}$ were analyzed. Log-rank p values are shown. **c** Control cells retrovirally transfected with or without estrogen receptor (ER)-$KRAS^{G12V}$ were treated with or without 0.1 μM of 4OHT for 3 days. The expression 4OHT-inducible ER-$KRAS^{G12V}$, endogenous pan-RAS and α-Tubulin were analyzed by western blotting. **d** Estimated cell number of Control cells treated with 0.1 μM of 4OHT for 28 days shown in (**1d**) with a different scale. The results represent the means ± SEM of three independent experiments. **e** Top, biological duplication of (**1e**). * The sample of control cells treated with 0.1 μM of 4OHT for 21 days was lost during preparations, Quantification of the relative ATR protein level normalized by each α-Tubulin protein level. The results represent the means ± SEM of (**1e & S1e**). two-tailed paired parametric t test. **f** The expression level of ATR mRNA in RSTCs #2, #5 and #7 maintained in 0.1 μM of 4OHT-containing medium were analyzed by SYBR Green RT-qPCR. The results represent the means ± SEM of three independent experiments. one-way ANOVA Tukey's test. **g** The expression level of miR185 in RSTCs #2, #5 and #7 maintained in 0.1 μM of 4OHT-containing medium were analyzed by TaqMan RT-qPCR. The results represent the means ± SEM of three independent experiments. one-way ANOVA Tukey's test. **h** Total copy number of ATR-encoded Chr3. Copy number gain of Chr3 was observed in RSTC#5 and #7. Blue arrow indicates ATR-encoded position. **i** Top, representative result of Cycloheximide (CHX) treatment assay of RSTCs #2, #5 cultured in 0.1 μM of 4OHT-containing medium. Cells were treated with 300 μg/ml of CHX for indicated times. The expression level of indicated proteins were analyzed by western blotting. Bottom, quantification of the relative ATR protein level normalized by each α-Tubulin protein level. The results represent the means ± SEM of three independent experiments. **j** Quantification of EdU positive cells in RSTCs #2, #5 and #7 maintained in 0.1 μM of 4OHT-containing medium were analyzed by EdU-Click iT assay. The results represent the means ± SEM of three independent experiments. one-way ANOVA Tukey's test. **k** top, representative result of Roscovitine (Rosc) treatment assay in Control, RSTCs #2, #5 and #7. Cells were treated with 25 μM of Rosc for 24 h. Bottom, quantification of the relative ATR protein level normalized by each α-Tubulin protein level. The results represent the means ± SEM of four independent experiments. one-way ANOVA Tukey's test. **l** Representative result of 4OHT free assay in RSTCs #2, #5 and #7. RSTC clones were split into with or without 0.1 μM of 4OHT conditions and cultured for 31 days. The expression level of indicated proteins were analyzed by western blotting. **m** Quantification of cell proliferation of the cells. Cell growth was determined using PrestoBlue after 0.1 μM of 4OHT treatment for 7 days. The results represent the means ± SEM of four independent experiments. one-way ANOVA Tukey's test. **n** The indicated proteins expression level in Control cells maintained with or without 0.1 μM of 4OHT-containing medium for ~14 days were analyzed by western blotting. Representative result of two independent reproducible experiments are shown. **o** The indicated proteins expression level in RSTC clones #2, #5 and #7 maintained in 0.1 μM of 4OHT-containing medium were analyzed by western blotting. Control and ATR-1 cells incubated for 3 days were shown as control. Representative result of three independent reproducible experiments are shown. All source data are provided as a Source Data file.

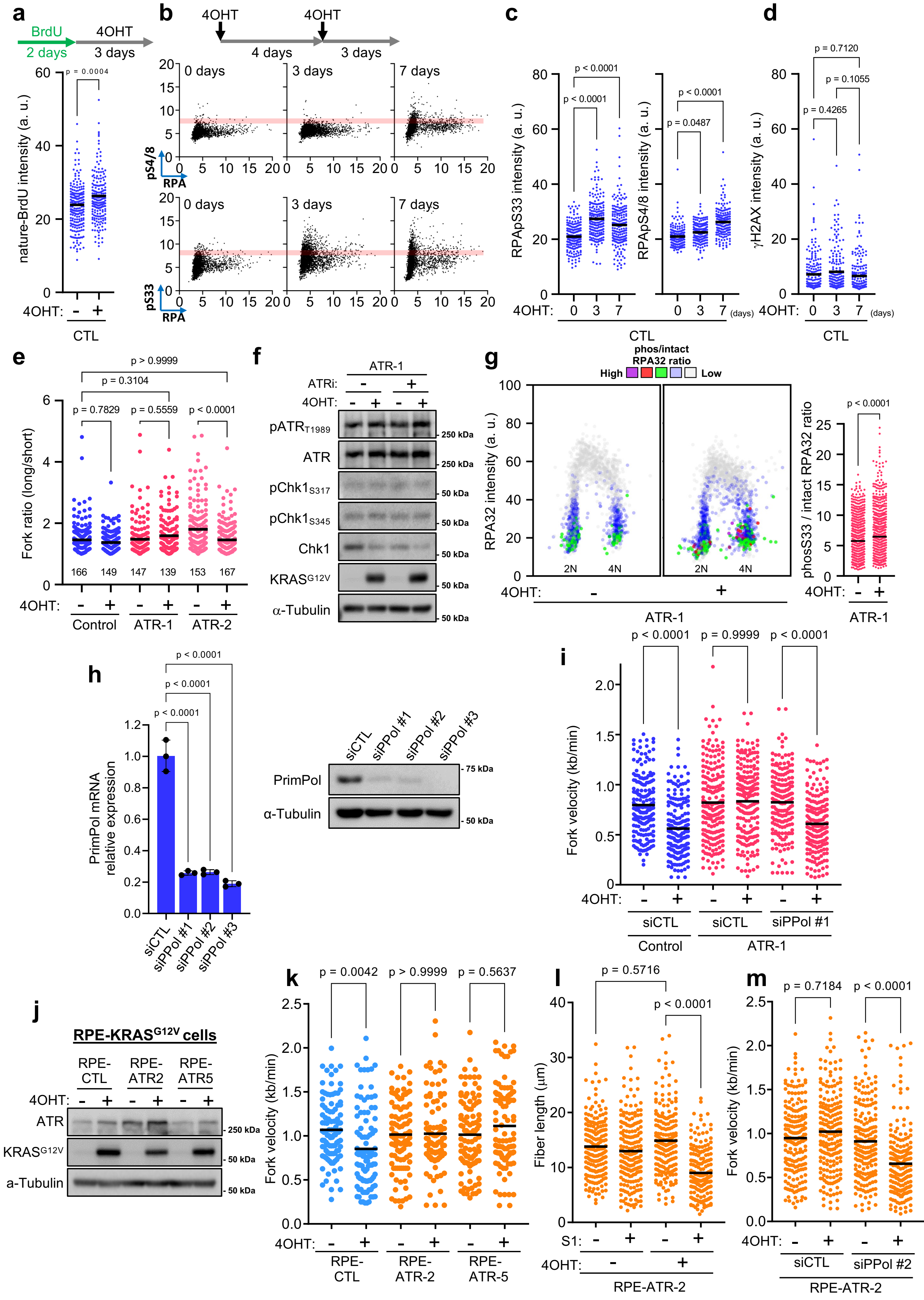

Igarashi et al. Supplementary Figure 2

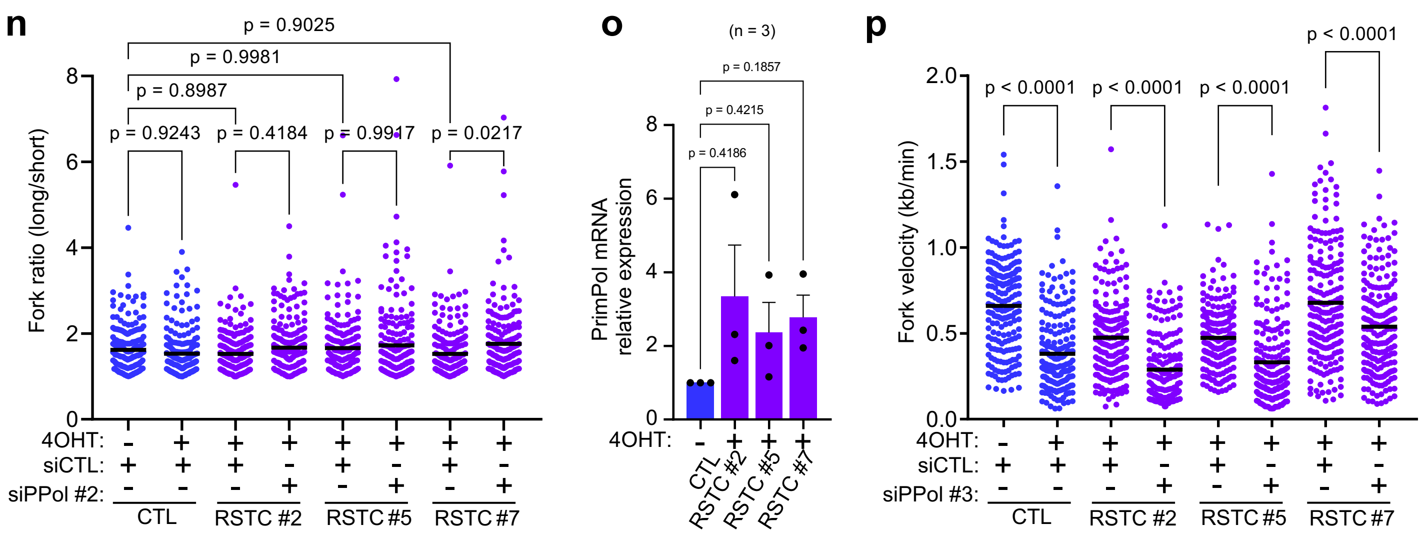

Supplementary Figure 2.

a Native-BrdU detection assay. Cells were grown in 10 μM of BrdU-containing medium and incubated for 2 days before released into BrdU-free medium with or without 0.1 μM of 4OHT for 3 days. Representative result of two independent reproducible experiments. Black lines indicate the mean; arbitrary units, a. u.; n = 200; two-tailed Mann-Whitney t test. **b, c** Cells were treated with 0.1 μM of 4OHT for 3 or 7 days respectively, then followed by immunofluorescence. Representative result of three independent reproducible experiments are shown. **b** Scatterplot shows the mean of intact RPA32 intensity (x axis) versus mean of phospho-RPA32 (Ser4/8 or Ser33) intensity (y axis). 3,000 cells are shown. **c** Quantification of the phospho-RPA32 (Ser4/8 or Ser33) intensity shown in (**S2b**). arbitrary units, a. u. **d** Quantification of the γH2AX intensity after 4OHT treatment for 3 or 7 days, respectively. Representative result of two independent reproducible experiments. arbitrary units, a. u. **e** Dot plot and mean of fork ratio in Control, ATR-1 and ATR-2 cells treated with 0.1 μM of 4OHT for 3 days shown in (**2b**). **f** Representative western blotting result of ATR-1 cells treated with 0.1 μM of 4OHT for 3 days shown in (**2c**). Low dose of ATRi (1 nM) was added 24 h prior to sampling. **g** Left, quantitative image-based cytometry (QIBC) of chromatin-bound phospho-RPA32 (Ser33) and intact-RPA32 visualized by pre-extraction method in ATR-1 cells after 0.1 μM of 4OHT treatment for 3 days. The color of 3,000 cells indicates phospho-RPA32 (Ser33) intensity normalized by its intact-RPA32 intensity. Right, quantification of normalized phospho-RPA32 (Ser33) intensity. Representative result of two independent reproducible experiments are shown. Black lines indicate the mean; n = 1000; two-tailed Mann-Whitney t test. **h** Control SAECs were transfected with 1 nM of three independent siRNAs of PrimPol for 24 h. The expression level of PrimPol were analyzed by SYBR Green RT-qPCR and western blotting. Data are presented as mean ± SD (n=3). one-way ANOVA Tukey's test. **i** Dot plot and mean of fork speed in Control SAECs and ATR-1 cells transfected with 1 nM of siControl or siPPol #1 for 24 h and treated with 0.1 μM of 4OHT for 3 days. Representative result of three independent reproducible experiments are shown. **j-m** RPE-derived Control, ATR-2 and ATR-5 cells were treated with or without 0.1 μM of 4OHT for 3 days. **j** The indicated proteins expression levels were analyzed by western blot analysis. **k** Dot plot and mean of fork speed. Representative result of three independent reproducible experiments are shown. Black lines indicate the mean; n = 100; one-way ANOVA Tukey's test. **l** Dot plot and mean of fiber lengths with or without 10 U/ml of S1 nuclease treatment for 30 min. Representative result of two independent reproducible experiments are shown. **m** Dot plot and mean of fork speed with 1 nM of siControl or siPPol #2 transfection for 24 h and 0.1 μM of 4OHT treatment for 3 days. Representative result of three independent reproducible experiments are shown. **n** Dot plot and mean of fork ratio in SAEC-parental cells, RSTCs #2, #5 and #7 transfected with 1 nM of siControl or siPPol #2 for 24 h and treated with 0.1 μM of 4OHT for 3 days as shown in (**2g**). **o** The mRNA expression level of PrimPol in RSTCs #2, #5 and #7 maintained in 0.1 μM of 4OHT-containing medium were analyzed by SYBR Green RT-qPCR. The results represent the means ± SEM of three independent experiments. one-way ANOVA Tukey's test. **p** Dot plot and mean of fork speed in control cells and RSTCs #2, #5 and #7 transfected with 1 nM of siControl or siPPol #3 for 24 h and treated with 0.1 μM of 4OHT for 3 days. Representative result of two independent reproducible experiments are shown. **c, d, e, i, l-n, p** Black lines indicate the mean; n = 200; one-way ANOVA Tukey's test. All source data are provided as a Source Data file.

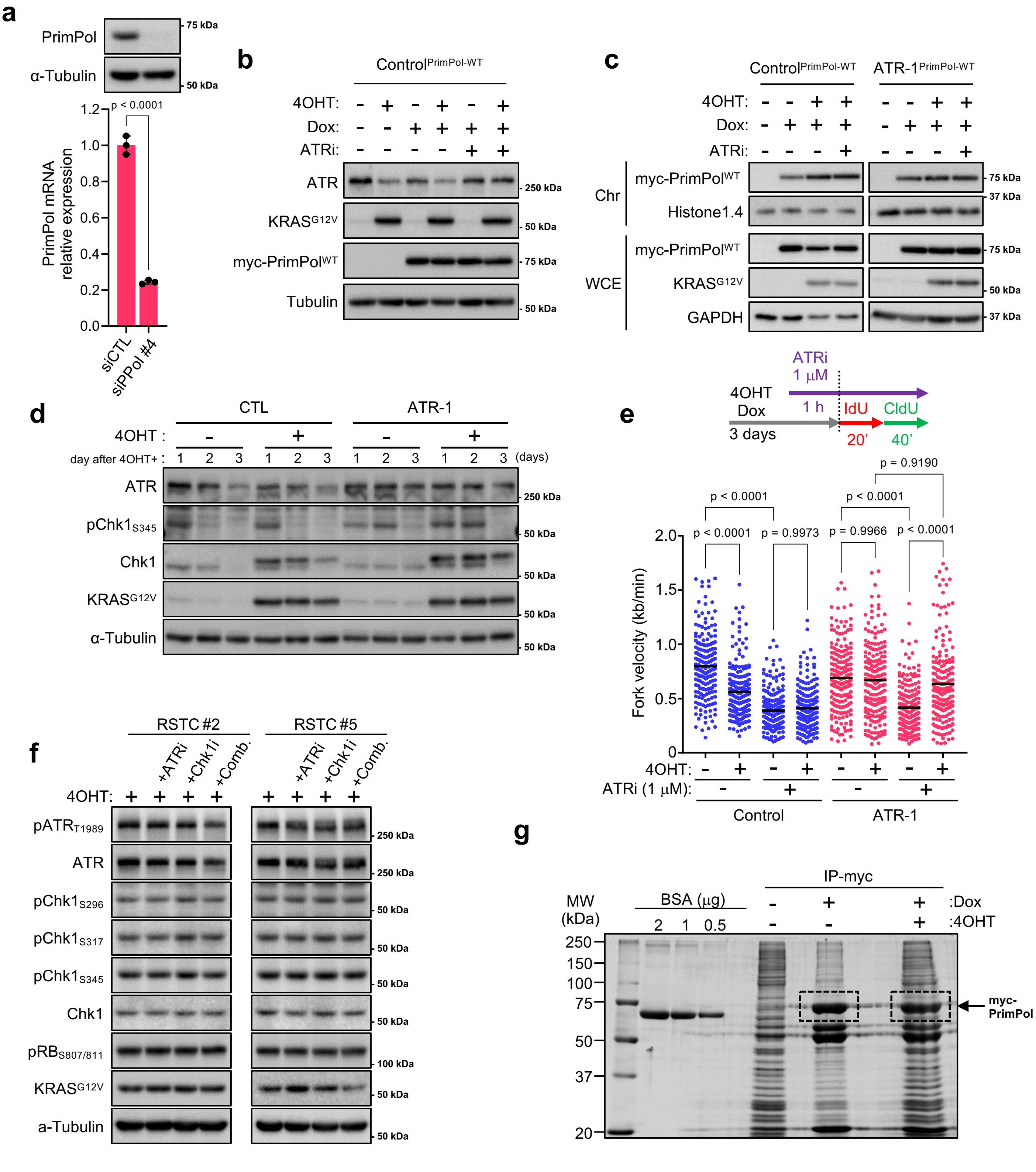

h

Gene names	Positions within proteins	Amino acid	Sequence window	Fold change (Log)
PRIMPOL	26	S	QIEERASHYERKPLSSVYRPRLSKPEEPPSI	1.74
	33	S	HYERKPLSSVYRPRLSKPEEPPSIWRLFHRQ	1.35
	207	S	ILQPALDLLGSEDDDSAPETTGHGFPHFSEA	-1.35
	255	S	EESWTSNSKKLERLGSAEQSSPDLSFLVVKN	1.21
	499	S	ADETRSNETQNPHKPSPSRLSTGASADAVWD	-1.31
	489, 499	S, S	ADETRSNETQNPHKPSPSRLSTGASADAVWD	-2.21

Supplementary Figure 3.

a ATR-1 cells were transfected with 5 nM of siUTR-PrimPol (siPPol #4) for 24 h. The mRNA expression level of PrimPol were analyzed by RT-qPCR. Data are presented as mean ± SD (n=3). two-tailed unpaired t test. **b** The indicated proteins expression level in Control cells harboring doxycycline-inducible myc-PrimPol^{WT} treated with 0.1 μM of 4OHT and with or without 1 μg/ml of doxycycline for 3 days were analyzed by western blotting. 1 nM of ATRi was added 24 h prior to sampling. Representative result of two independent reproducible experiments are shown. **c** Representative result of two independent reproducible chromatin fractionation assay. Control and ATR-1 cells harboring doxycycline-inducible myc-PrimPol^{WT} treated with 0.1 μM of 4OHT and with or without 1 μg/ml of doxycycline for 3 days. 1 nM of ATRi was added 24 h prior to sampling. The indicated protein levels were analyzed by western blotting. **d** Representative result of four independent reproducible time-chase assay. The indicated proteins expression level in Control and ATR-1 cells treated with 0.1 μM of 4OHT for 1~3 days were analyzed by western blotting. **e** Dot plot and mean of fork speed in Control and ATR-1 cells treated with 0.1 μM of 4OHT for 3 days. High dose of ATRi (1 μM) was added 1 h prior to IdU/CldU labeling. Representative result of two independent reproducible experiments are shown. Black lines indicate the mean; n = 200; one-way ANOVA Tukey's test. **f** The indicated proteins expression level in RSTC #2 and #5 treated with 0.1 μM of 4OHT for 3 days were analyzed by western blotting. Low dose of ATRi (1 nM), Chk1i (1 nM) and those combination (Comb.) was added 24 h prior to sampling. Representative result of two independent reproducible experiments are shown. **g, h** Identification of phosphorylated site of PrimPol with LC-MS/MS. **g** The result of SYPRO Ruby Protein Gel stain for phospho-proteomics sampling. ATR-1 cells harboring doxycycline-inducible myc-PrimPol^{WT} were treated with 0.1 μM of 4OHT and with or without 1 μg/ml of doxycycline for 3 days. Representative result of three independent reproducible experiments are shown. **h** The list of phosphorylated or dephosphorylated site (highlighted in red) of myc-PrimPol revealed by phospho-proteomics analysis. All source data are provided as a Source Data file.

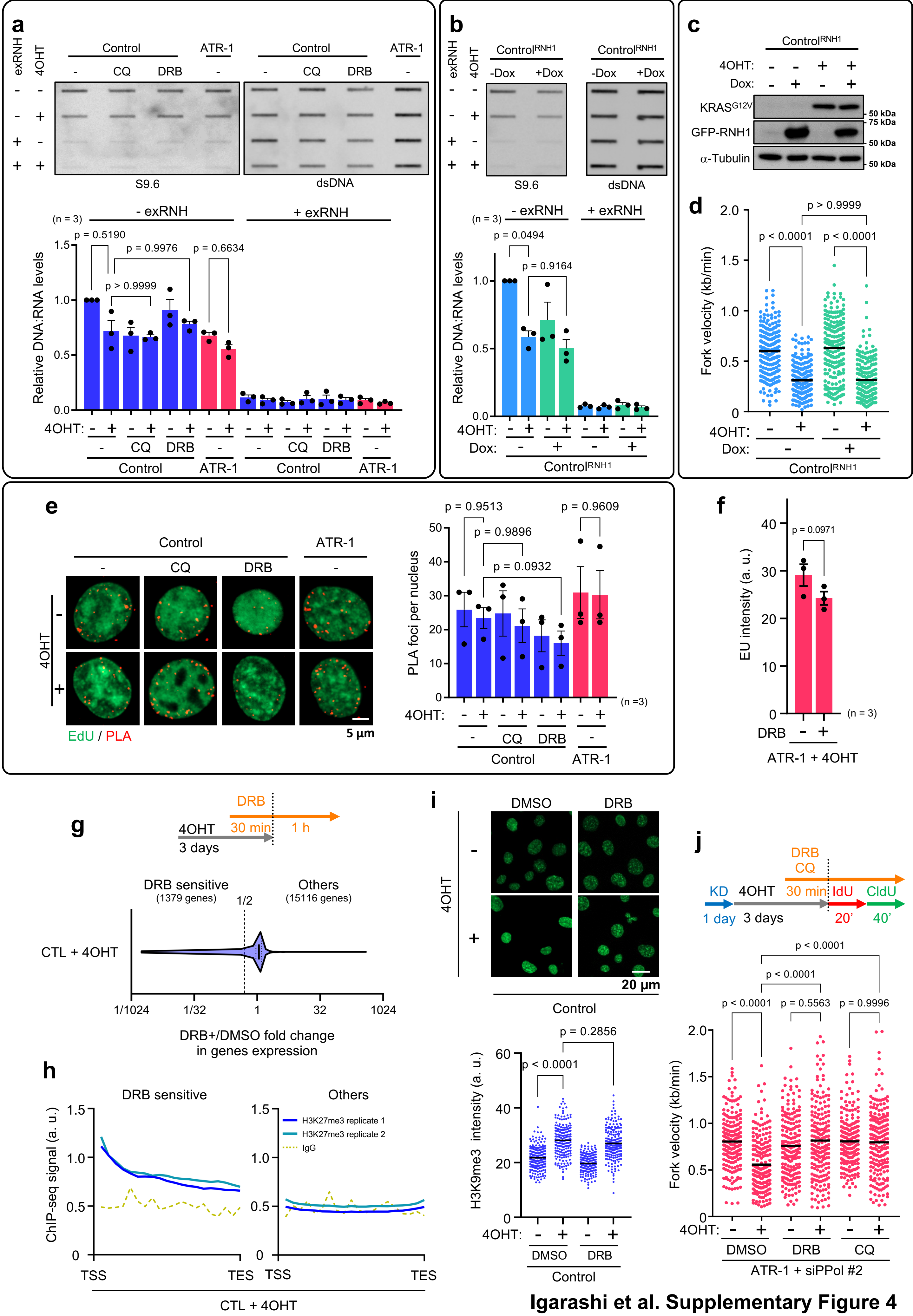

Igarashi et al. Supplementary Figure 4

Supplementary Figure 4.

a Top, representative result of slot blot assay for R-loop. Control and ATR-1 cells were treated with 0.1 μM of 4OHT for 3 days. 100 μM of DRB or 40 μM of Chloroquine (CQ) was added 90 min prior to sampling. Bottom, quantification of the S9.6 signal normalized by parallel dsDNA signal. The results represent the means ± SEM of three independent experiments. one-way ANOVA Tukey's test. **b** Top, representative result of slot blot assay for R-loop. Control cells harboring doxycycline-inducible GFP-RNaseH1 were treated with 0.1 μM of 4OHT and with or without 1 μg/ml of doxycycline for 3 days. Bottom, quantification of the S9.6 signal normalized by parallel dsDNA signal. The results represent the means ± SEM of three independent experiments. one-way ANOVA Tukey's test. **c, d** Control cells harboring doxycycline-inducible GFP-RNaseH1 were treated with 0.1 μM of 4OHT with or without 1 μg/ml of doxycycline for 3 days. **c** The indicated proteins expression level were analyzed by western blotting. **d** Dot plot and mean of fork speed. Representative result of two independent reproducible experiments are shown. **e** Left, representative proximity ligation assay (PLA) foci image showing transcription-replication collision (TRC) in Control and ATR-1 cells treated with 0.1 μM of 4OHT for 3 days. 100 μM of DRB or 40 μM of CQ was added 90 min prior to fixation. Cells were treated with 10 μM of EdU for final 30 min to detect S-phase, then PLA was performed using antibody against PCNA and phospho-POLII (Ser2). Right, number of PLA foci in EdU positive cells. The results represent the means ± SEM of three independent experiments. one-way ANOVA Tukey's test. **f** Quantification of EU intensity of ATR-1 cells treated with 0.1 μM of 4OHT for 3 days. 1 mM of EU was added 23 h prior to 100 μM of DRB treatment for final 60 min. The results represent the means ± SEM of three independent experiments. two-tailed paired parametric t test. **g** Quantification of fold change (FC) in gene expression with DRB treatment analyzed by RNA-seq. Control cells were treated with 0.1 μM of 4OHT for 3 days and 100 μM of DRB treatment for totally 90 min (see **4a**). FC<1/2 genes were determined as DRB sensitive. **h** The distribution of H3K27me3 ChIP-seq signals within gene loci were visualized by the scale-regions function of deepTools. H3K27me3 ChIP-seq data of SAEC were provided from Suzuki, A. et al. (PMID: 25378332). arbitrary units, a. u. **i** Top, representative image of chromatin-bound H3K9me3 staining. After 0.1 μM of 4OHT treatment for 3 days, control cells were treated with 100 μM of DRB for 90 min, followed by staining with anti-H3K9me3 antibody with pre-extraction method. Scale bar=20 μm. Bottom, quantification of the H3K9me3 intensity. Representative result of two independent reproducible experiments are shown. arbitrary units, a. u. **j** Dot plot and mean of fork speed in ATR-1 cells transfected with 1 nM of siPPol #2 for 24 h and treated with 0.1 μM of 4OHT for 3 days. 100 μM of DRB or 40 μM of CQ was added 30 min prior to IdU/CldU labeling. Representative result of four independent reproducible experiments are shown. **d, i, j** Black lines indicate the mean; n = 200; one-way ANOVA Tukey's test. All source data are provided as a Source Data file.

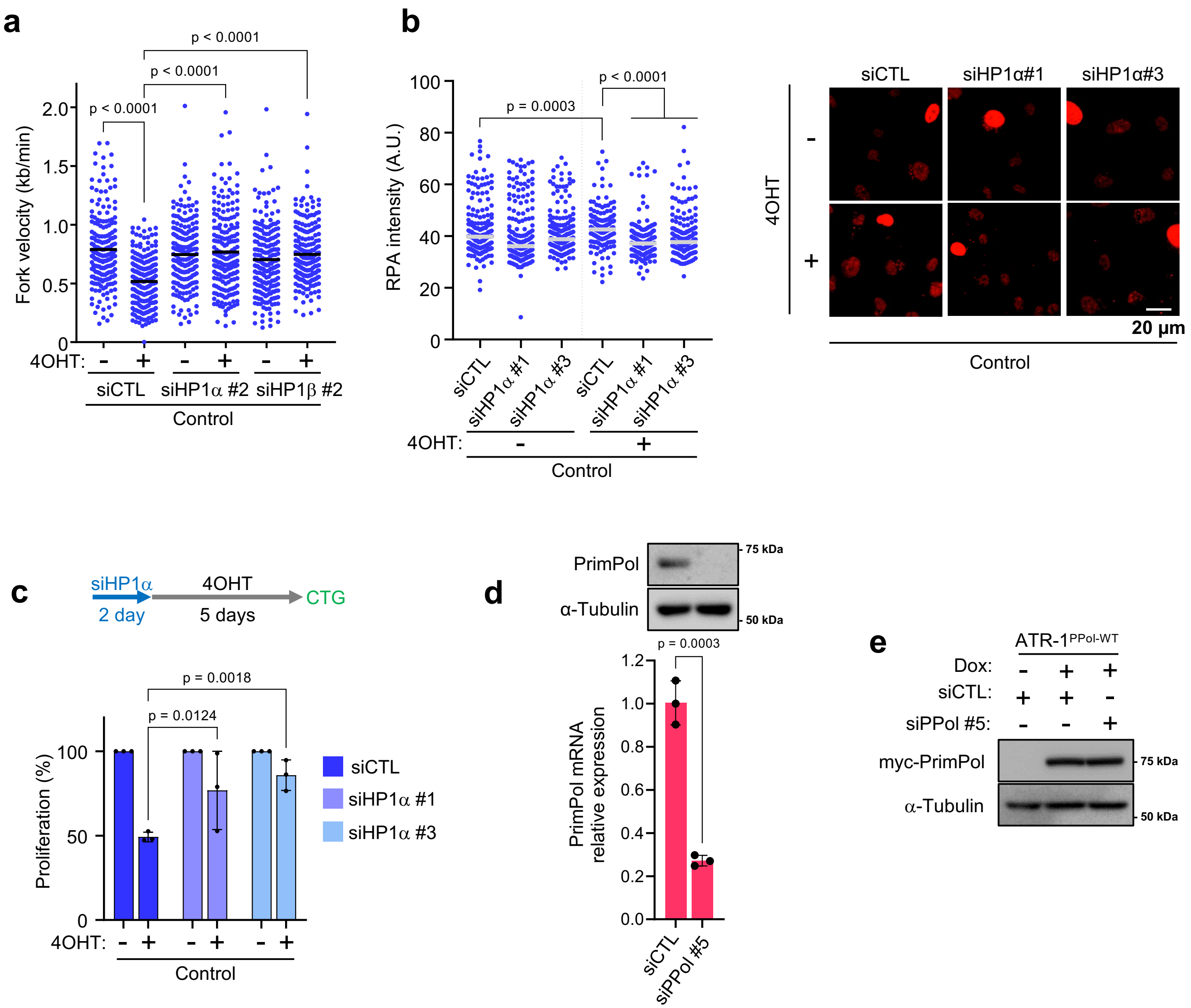

Supplementary Figure 5.

a Dot plot and mean of fork speed in Control cells transfected with 1 nM of siControl, siHP1α #2 or siHP1β #2 for 24 h and treated with 0.1 μM of 4OHT for 3 days. Representative result of two independent reproducible experiments are shown. **b** Left, Quantification of the RPA32 intensity. Control cells were transfected with 1 nM of siControl, siHP1α #1 or siHP1α #3 for 24 h, then treated with 0.1 μM of 4OHT for 3 days. Representative result of two independent reproducible experiments are shown. Gray lines indicate the median; n = 200; two-tailed Mann-Whitney t test. Right, Representative image of RPA immunofluorescence. Scale bar=20 μm. **c** Quantification of cell proliferation of the cells. Cells were transfected with 1 nM of siControl or siHP1α #1,3 for 48 h and incubated with 0.1 μM of 4OHT for 5 days. Cell growth was determined using Cell Titer-Glo 2.0 Assay. The results represent the means ± SEM of three independent experiments. two-way ANOVA Šídák's multiple comparisons test. **d** ATR-1 cells were transfected with 1 nM of siControl and siUTR-PrimPol (siPPol #5) for 24 h. The expression level of PrimPol were analyzed by RT-qPCR and Western Blotting. Data are presented as mean ± SD (n=3). two-tailed unpaired t test. **e** ATR-1 cells harboring doxycycline-inducible myc-PrimPol^{WT} were transfected with 1 nM of siControl or siPPol #5 for 24 h and treated with 1 μg/ml of doxycycline for 24 h. The expression level of myc-tag PrimPol and α-Tubulin were analyzed by western blotting. **a, b** Black lines indicate the mean; n = 200; one-way ANOVA Tukey's test. All source data are provided as a Source Data file.

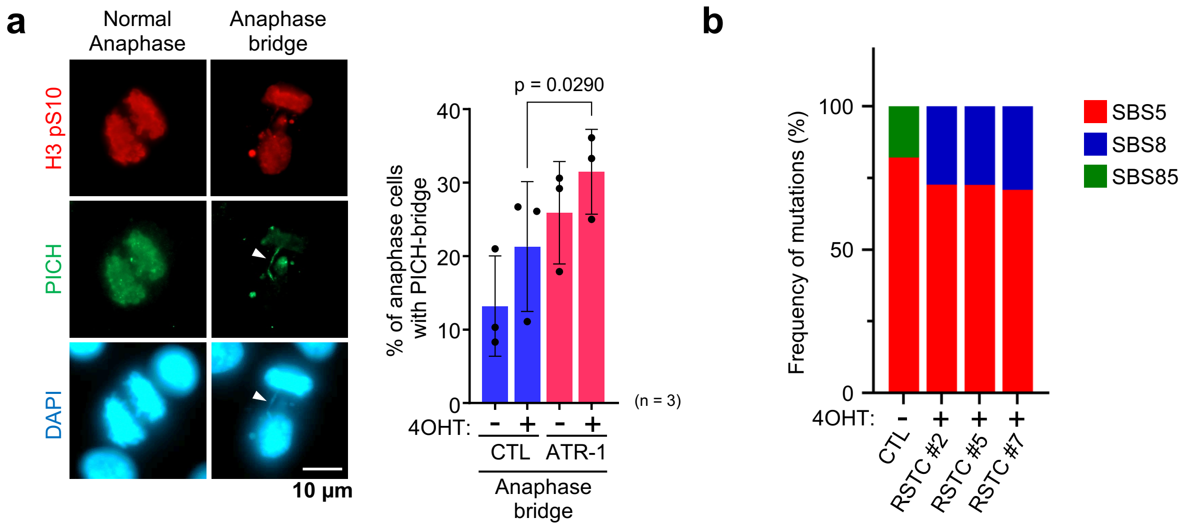

Supplementary Figure 6.

a Left, representative image of phospho-HistoneH3 (Ser10), PICH and DAPI co-staining. White arrow indicates the anaphase bridge. Right, quantification of the percentage of anaphase bridge-positive cells in M-phase cells. The results represent the means ± SEM of three independent experiments. one-way ANOVA Tukey's test. **b** The frequency of mutations in RSTC#2, #5 and #7 assigned to signatures SBS5, SBS8 and SBS85 are shown. All source data are provided as a Source Data file.

a

Cell line	Driver Gene	Driver aberration
H1975	EGFR	L858R / T790M
H1819	ERBB	amp.
H3122	EML4-ALK	fusion
A427	KRAS	G12D
H358	KRAS	G12C
H2009	KRAS	G12A

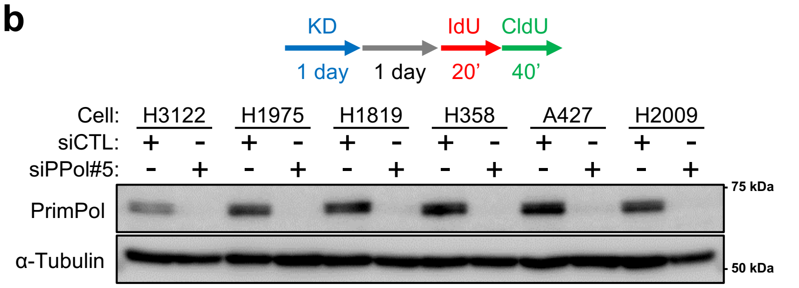

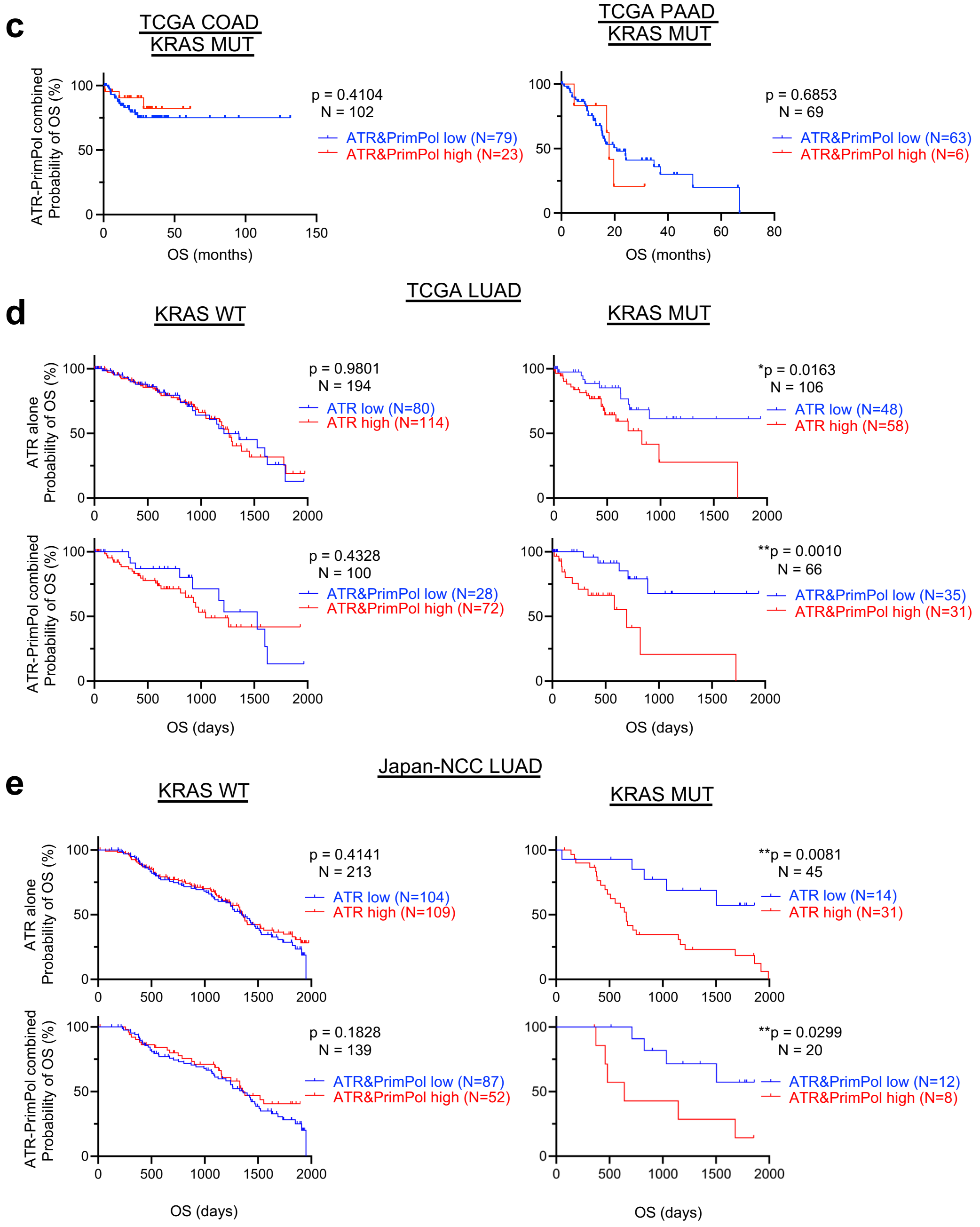

Supplementary Figure 7.

a The character of KRAS^{WT} cells (H3122, H1975, H1819) and KRAS^{G12mut} cells (H358, A427, H2009). The information was obtained from *Cellosaurus* (PMID: 29805321). **b** Cells were transfected with 1 nM of siControl or siPPol #5 for 24 h and released into siRNA free-medium for 24 h. The expression level of PrimPol and α-Tubulin were analyzed by western blotting. Representative result of two independent reproducible experiments are shown. **c** OS according to ATR mRNA expression and PrimPol mRNA from COAD and PAAD patients harboring KRAS^{Mut} were analyzed. Log-rank p values are shown. **d** TCGA-cohort analysis. OS according to ATR and PrimPol mRNA expression from LUAD patients, who experienced smoking, harboring KRAS^{WT} and KRAS^{Mut} were analyzed. **e** NCC (Japan) cohort analysis. OS according to ATR and PrimPol mRNA expression from LUAD patients, who experienced smoking, harboring KRAS^{WT} and KRAS^{Mut} were analyzed. All source data are provided as a Source Data file.
