# Supplementary material for: An ATR-PrimPol pathway confers tolerance to oncogenic KRAS-induced and heterochromatin-associated replication stress
Source: Nat Commun. 2023 Aug 17;14:4991. doi: 10.1038/s41467-023-40578-2 (PMC10435487; doi:10.1038/s41467-023-40578-2)
Supplement: Supplementary file 3 — Reporting Summary [file 41467_2023_40578_MOESM3_ESM.pdf]

nature portfolio

Corresponding author(s): Bunsyo Shiotani

Last updated by author(s): Jul 10, 2023

Reporting Summary

Nature Portfolio wishes to improve the reproducibility of the work that we publish. This form provides structure for consistency and transparency in reporting. For further information on Nature Portfolio policies, see our Editorial Policies and the Editorial Policy Checklist.

Statistics

For all statistical analyses, confirm that the following items are present in the figure legend, table legend, main text, or Methods section.

n/a	Confirmed	
☐	☒	The exact sample size (*n*) for each experimental group/condition, given as a discrete number and unit of measurement
☐	☒	A statement on whether measurements were taken from distinct samples or whether the same sample was measured repeatedly
☐	☒	The statistical test(s) used AND whether they are one- or two-sided<br>*Only common tests should be described solely by name; describe more complex techniques in the Methods section.*
☐	☒	A description of all covariates tested
☐	☒	A description of any assumptions or corrections, such as tests of normality and adjustment for multiple comparisons
☐	☒	A full description of the statistical parameters including central tendency (e.g. means) or other basic estimates (e.g. regression coefficient) AND variation (e.g. standard deviation) or associated estimates of uncertainty (e.g. confidence intervals)
☐	☒	For null hypothesis testing, the test statistic (e.g. *F*, *t*, *r*) with confidence intervals, effect sizes, degrees of freedom and *P* value noted<br>*Give P values as exact values whenever suitable.*
☒	☐	For Bayesian analysis, information on the choice of priors and Markov chain Monte Carlo settings
☒	☐	For hierarchical and complex designs, identification of the appropriate level for tests and full reporting of outcomes
☒	☐	Estimates of effect sizes (e.g. Cohen's *d*, Pearson's *r*), indicating how they were calculated

Our web collection on statistics for biologists contains articles on many of the points above.

Software and code

Policy information about availability of computer code

Data collection	1. Immunoblot / Gel staining / Colony staining were imaged using LAS 3000 luminescent image analyzer (Fujifilm).<br>2. DNA fiber / Immunofluorescence / PLA / Micro Nuclei staining were imaged using Celldiscoverer 7 (Zeiss) and/or TCS SP8 (Leica).<br>3. WGS library was sequenced on NovaSeq 6000 system (Illumina).<br>4. RT-qPCR data was obtained using StepOnePlus thermocycler (Applied Biosystem).<br>5. LC-MS/MS data was obtained using Q Exactive hybrid quadrupole-Orbitrap mass spectrometer (Thermo Fisher Scientific).<br>6. RNA-seq data was obtained using DNBSEQ-G400 (MGI tech).
Data analysis	Prism v9.5.0, ImageJ v2.0.0-rc-69/1.52p, ZEN v2.6 and/or v3.1, BWA-MEM v0.7.17, gatk2 v 4.1.2.0, Manta program v1.6.0, shinyCircos program v1, FACETS v0.6.2, SigprofilerMatrixGenerator v1.2.12, SigprofilerExtracter v1.1.2, COSMIC signatures v3.3, MaxQuant v1.6.14.0, FastQC software (version XX), Kallisto v0.46.0, R software v3.6.3

For manuscripts utilizing custom algorithms or software that are central to the research but not yet described in published literature, software must be made available to editors and reviewers. We strongly encourage code deposition in a community repository (e.g. GitHub). See the Nature Portfolio guidelines for submitting code & software for further information.

Data

Policy information about availability of data

All manuscripts must include a data availability statement. This statement should provide the following information, where applicable:

- Accession codes, unique identifiers, or web links for publicly available datasets
- A description of any restrictions on data availability
- For clinical datasets or third party data, please ensure that the statement adheres to our policy

RNA-seq data from SAEC cell samples generated in this study have been deposited in the NCBI GEO database under accession code GSE223027. ChIP-seq data from SAEC cell samples used in this study have been deposited in the DDBJ data base under accession code DRA002311. Proteomics data generated in this study have been deposited in JPOST, a public proteome database certified by the ProteomeXchange Consortium, under accession code PXD043419 (Access key:2101)
WGS data generated in this study have been deposited in DDBJ under accession code "PRJDB16238[https://ddbj.nig.ac.jp/resource/bioproject/PRJDB16238]".
UniProt human database is available at (https://www.uniprot.org). Human genome (GRCh38) data is available at (https://gdc.cancer.gov/about-data/gdc-data-processing/gdc-reference-files). The Whole Transcriptome Sequencing (WTS) data from NCCJ-cohort that support the findings of this study are not publicly available and restrictions apply to the availability of these data. Such WTS data are available through to the corresponding authors (Bunsyo Shiotani: bshiotan@ncc.go.jp) for academic non-commercial research purposes upon reasonable request, and subject to review of a project proposal that will be evaluated by PRISM data access committee, entering into an appropriate data access agreement and subject to any applicable ethical approvals. The data supporting the findings of this study are available within the Article and its supplementary information files or Source Data files. Source data are provided with this paper.

Human research participants

Policy information about studies involving human research participants and Sex and Gender in Research.

Reporting on sex and gender	Analysis were performed without considering sex and gender.
Population characteristics	Lung cancer patients
Recruitment	National Cancer Center BioBank.
Ethics oversight	National Cancer Center Institutional review board

Note that full information on the approval of the study protocol must also be provided in the manuscript.

Field-specific reporting

Please select the one below that is the best fit for your research. If you are not sure, read the appropriate sections before making your selection.

☒ Life sciences ☐ Behavioural & social sciences ☐ Ecological, evolutionary & environmental sciences

For a reference copy of the document with all sections, see nature.com/documents/nr-reporting-summary-flat.pdf

Life sciences study design

All studies must disclose on these points even when the disclosure is negative.

Sample size	No statistical method was used to predict sample size. The sample size was guided by preliminary studies we carried out, and guided by a number of previous publications using similar approaches. Sample size was kept similar amongst different conditions.<br>Sample size for all experiments shown (electron microscopy, n>200 or 1000 in 3 independent experiments; DNA fibers, n>200 in 2 or more independent experiments for IdU/CldU ratio or tract lengths, n>200 in 2 or more independent experiments for sister fork asymmetry; micronuclei, n>200 in 3 or more independent experiments; QIBC, >3000 in 3 or more independent experiments) was chosen to obtain statistical power. When the sample size is less than indicated above, we have provided n numbers in the figure legends.<br>For anchorage-independent growth assay, all of the stained colonies were counted in 3 or more independent experiments.
Data exclusions	No data was excluded.
Replication	All experiments were biologically repeated at least twice, at most six times. Each data details were shown in figure legends.
Randomization	The experiments were not randomized. We used cell lines only for all experiments. Randomization is not generally used for experiments except for DNA fiber/Immunofluorescence/PLA assay. Obtained raw data of DNA fiber / Immunofluorescence / PLA were randomly selected.
Blinding	Blinding was not relevant to this study because analyses were analyst independent.

Reporting for specific materials, systems and methods

We require information from authors about some types of materials, experimental systems and methods used in many studies. Here, indicate whether each material, system or method listed is relevant to your study. If you are not sure if a list item applies to your research, read the appropriate section before selecting a response.

Materials & experimental systems

n/a	Involved in the study
☐	☒ Antibodies
☐	☒ Eukaryotic cell lines
☒	☐ Palaeontology and archaeology
☒	☐ Animals and other organisms
☒	☐ Clinical data
☒	☐ Dual use research of concern

Methods

n/a	Involved in the study
☒	☐ ChIP-seq
☒	☐ Flow cytometry
☒	☐ MRI-based neuroimaging

Antibodies

Antibodies used

anti-pan-RAS (WB 1:1000, Santa Cruz, Cat# sc-166691), anti-αTubulin (WB 1:5000, MBL, Cat# PM054), anti-phospho-ATR (Thr1989) (WB 1:3000, Cell Signaling Technology, Cat# 30632), anti-ATR (WB 1:3000, GeneTex, Cat# GTX128146), anti-phospho-Chk1 (Ser317) (WB 1:500, Cell Signaling Technology, Cat# 12302), anti-phospho-Chk1 (Ser345) (WB 1:500, Cell Signaling Technology, Cat# 2348), anti-Chk1 (WB 1:100, Santa Cruz, Cat# sc-8408), anti-phospho-RB (Ser807/811) (WB 1:1000, Cell Signaling Technology, Cat# 8516), anti-E-cadherin (WB 1:1000, Cell Signaling Technology, Cat# 3195), anti-Vimentin (WB 1:1000, Cell Signaling Technology, Cat# 5741), anti-PrimPol (WB 1:3000, Proteintech, Cat# 29824-1-AP), anti-myc tag (WB 1:1000, MBL, Cat# 192-3), anti-HistoneH1.4 (WB 1:1000, Cell Signaling Technology, Cat# 41328), anti-GAPDH (WB 1:1000, Wako, Cat# 010-25521), anti-phospho-Chk1 (Ser296) (WB 1:500, Cell Signaling Technology, Cat# 90178), anti-RNaseH1 (WB 1:1000, Abcam, Cat# ab56560), anti-HP1α (WB 1:1000, Millipore, Cat# 05-689), anti-HP1β (WB 1:1000, Cell Signaling Technology, Cat# 8676), Peroxidase-conjugated AffiniPure Goat Anti-Rabbit or Mouse IgG (H+L) (WB 1:5000, Jackson ImmunoResearch, Cat# 111-035-003 or Cat# 115-035-003), anti-IdU (DNA fiber assay 1:25, BD Biosciences, 347580), anti-CldU (DNA fiber assay 1:100, Abcam, ab6326), Alexa Fluor 488-conjugated anti-rat IgG (DNA fiber assay 1:500, Invitrogen, Cat# A11006), Alexa Fluor 555-conjugated anti-mouse IgG (DNA fiber assay 1:250, Invitrogen, Cat# A21422), anti-BrdU (IF 1:1000, PLA 1:1000, GE health, Cat# RPN20AB), anti-RPA32 (IF 1:200, Abcam, Cat# ab2175), anti-phospho-RPA32 (Ser4/8) (IF 1:200, Bethyl, # A300-245A), anti-phospho-RPA32 (Ser33) (IF 1:200, Bethyl, # A300-246A), anti-gH2AX (IF 1:500, Cell Signaling Technology, Cat# 9718), anti-H3K27me3 (IF 1:500, PLA 1:1600, Cell Signaling Technology, Cat# 9733), anti-H3K9me3 (IF 1:500, Abcam, Cat# ab8898), anti-PICH (IF 1:250, Abnova, Cat# H00054821-B01P), and anti-phospho-HistoneH3 (Ser10) (IF 1:500, Millipore, Cat# 06-570), Alexa 488 donkey anti-rabbit or anti-mouse IgG (H+L) (IF 1:500, Jackson ImmunoResearch, Cat# 711-545-152 or Cat# 715-545-151), Alexa 594 donkey anti-rabbit or anti-mouse IgG (H+L) (IF 1:500, Jackson ImmunoResearch, Cat# 711-585-152 or Cat# 715-585-151), anti-DNA-RNA hybrid clone S9.6 (SB 1:500, Millipore, Cat# MABE1095), anti-dsDNA (SB 1:5000, Abcam, Cat# ab27156), anti-PCNA (PLA 1:500, Santa Cruz, Cat# sc-56), anti-phospho-POLII (Ser2) (PLA 1:10000, Novus, Cat# NB100-1805)

Validation

anti-pan-RAS (Santa Cruz, Cat# sc-166691)
Species Reactivity: Human, Mouse, Rat
Validated applications: WB, IP, IF, IHC(P), ELISA

anti-αTubulin (MBL, Cat# PM054)
Species Reactivity: Human, Mouse, Rat, Hamstar, Chicken, Fruit fly
Validated applications: WB, IP, IC

anti-phospho-ATR (Thr1989) (Cell Signaling Technology, Cat# 30632)
Species Reactivity: Human
Validated applications: WB

anti-ATR (GeneTex, Cat# GTX128146)
Species Reactivity: Human, Mouse
Validated applications: WB, ICC/IF, IHC-P, IP

anti-phospho-Chk1 (Ser317) (Cell Signaling Technology, Cat# 12302)
Species Reactivity: Human, Mouse, Monkey
Validated applications: WB, IP, IF

anti-phospho-Chk1 (Ser345) (Cell Signaling Technology, Cat# 2348)
Species Reactivity: Human, Mouse, Rat, Monkey
Validated applications: WB, IF, FCM

anti-Chk1 (Santa Cruz, Cat# sc-8408)
Species Reactivity: Human, Mouse, Rat
Validated applications: WB, IP, IF, IHC(P), FCM, ELISA

anti-phospho-RB (Ser807/811) (Cell Signaling Technology, Cat# 8516)
Species Reactivity: Human, Mouse, Rat, Monkey

Validated applications: WB, IP, IHC, IF, FCM

anti-E-cadherin (Cell Signaling Technology, Cat# 3195)
Species Reactivity: Human, Mouse
Validated applications: WB, IHC, IF, FCM

anti-Vimentin (Cell Signaling Technology, Cat# 5741)
Species Reactivity: Human, Mouse, Rat, Monkey
Validated applications: WB, IHC, IF, FCM

anti-PrimPol (Proteintech, Cat# 29824-1-AP)
Species Reactivity: Human
Validated applications: WB, IHC, IF

anti-myc tag (MBL, Cat# 192-3)
Validated applications: WB, IP, FCM, IC, Co-IP, ChIP

anti-HistoneH1.4 (Cell Signaling Technology, Cat# 41328)
Species Reactivity: Human, Monkey
Validated applications: WB, IF, ChIP

anti-GAPDH (Wako, Cat# 010-25521)
Species Reactivity: Human, Mouse, African green monkey
Validated applications: WB, IP

anti-phospho-Chk1 (Ser296) (Cell Signaling Technology, Cat# 90178)
Species Reactivity: Human, Mouse, Rat
Validated applications: WB

anti-RNaseH1 (Abcam, Cat# ab56560)
Species Reactivity: Human
Validated applications: WB, FCM

anti-HP1α (Millipore, Cat# 05-689)
Species Reactivity: Human, Mouse, Vertebrates
Validated applications: WB, ICC, IHC, IP, ChIP

anti-HP1β (Cell Signaling Technology, Cat# 8676)
Species Reactivity: Human, Mouse, Rat, Monkey
Validated applications: WB, IP, IF, ChIP, CUT&RUN

anti-IdU (BD Biosciences, 347580)
Validated applications: FCM

anti-CldU (Abcam, ab6326)
Validated applications: ICC/IF, FCM, IHC-P

anti-BrdU (GE health, Cat# RPN20AB)
Validated applications: ICC

anti-RPA32 (Abcam, Cat# ab2175)
Species Reactivity: Human
Validated applications: FCM, WB, IHC-P

anti-phospho-RPA32 (Ser4/8) (Bethyl, # A300-245A)
Species Reactivity: Human, Mouse
Validated applications: ICC-IF, IHC, IP, WB

anti-gH2AX (Cell Signaling Technology, Cat# 9718)
Species Reactivity: Human, Mouse, Rat, Monkey
Validated applications: WB, IHC, IF, FCM

anti-H3K27me3 (Cell Signaling Technology, Cat# 9733)
Species Reactivity: Human, Mouse, Rat, Monkey
Validated applications: WB, IHC, IF, FCM, ChIP, CUT&RUN

anti-H3K9me3 (Abcam, Cat# ab8898)
Species Reactivity: Human, Mouse, Cow
Validated applications: WB, IHC-P, ICC/IF, ChIP

anti-PICH (Abnova, Cat# H00054821-B01P)
Species Reactivity: Human
Validated applications: WB

anti-phospho-HistoneH3 (Ser10) (Millipore, Cat# 06-570)
Species Reactivity: Human, Mouse

Validated applications: WB, ICC, IP

Anti-DNA-RNA hybrid clone S9.6 (Millipore, Cat# MABE1095)
Validated applications: ABA, ChIP, DB, ICC, IP

anti-dsDNA (Abcam, Cat# ab27156)
Species Reactivity: Human
Validated applications: IHC-P

anti-PCNA (Santa Cruz, Cat# sc-56)
Species Reactivity: Human, Mouse, Rat, Insect, S. pombe
Validated applications: WB, IP, IF, IHC(P), FCM

anti-phospho-POLII (Ser2) (Novus, Cat# NB100-1805)
Species Reactivity: Human, Mouse, Yeast, Drosophilia
Validated applications: WB, IHC, IHC-P, IP, Single-Cell Western

Eukaryotic cell lines

Policy information about cell lines and Sex and Gender in Research

Cell line source(s)	Human: HEK293T (ATCC, Cat# CRL-3216, RRID: CVCL_0063)<br>Human: SAEC (Lonza, Cat# CC-2547, Gift from Dr. Kiyono at National Cancer Center, Japan)<br>Human: RPE-1 (ATCC, Cat# CRL-4000, RRID: CVCL_4388, Gift from Dr. Hochegger at University of Sussex, UK)<br>Human: H3122 (obtained from NCI, RRID: CVCL_5160, Gift from Dr. Kobayashi, National Cancer Center, Japan,) (Confirmed by STR in 2023)<br>Human: H1975 (ATCC, Cat# CRL-5908, RRID: CVCL_1511)<br>Human: H1819 (ATCC, Cat# CRL-5897, RRID: CVCL_1497)<br>Human: H358 (ATCC, Cat# CRL-5807, RRID: CVCL_1559)<br>Human: A427 (ATCC, Cat# HTB-53, RRID: CVCL_1055)<br>Human: H2009 (ATCC, Cat# CRL-5911, RRID: CVCL_1514)
Authentication	H3122 was authenticated. The other cell lines were not authenticated.
Mycoplasma contamination	All cell lines are free of Mycoplasma contamination. We routinely do mycoplasma testing on our cell lines.
Commonly misidentified lines<br>(See ICLAC register)	Not used in this study.
